# What Exercise Does

**Published:** 1890-10

**Authors:** 


### WHAT EXERCISE DOES.

The following as to the value of systematic exercise in the gymnasium is from a young man who needed body-building. It is told in the *Magnet*, of the Springfield, Mass., association :

Five years ago, my health was in a deplorable condition. My friends said I couldn't live a year. I had been for years gulping down patent compounds of logwood, whiskey, avarice, and gall (the two latter ingredients being " pure and unadulterated "), when I suddenly awoke to the realization of the fact that if my life was to be prolonged, and my health restored, I had got to take a hand at the reconstruction business myself. I threw my medicine bottles and their contents into the gutter, and commenced a system of diet and exercise which I have since gradually added to and improved upon. It is a system that common sense would seem to dictate to any young man. To-day, I weigh more than I ever did before in my life, am stronger, feel better, and have an appetite that never fails me. True, I am not yet a well man, but all I ask for is time. It takes longer to rebuild than it does to tear down. Gymnasium work, out-door exercise, hunting and fishing, are doing for me what medical science, I believe, never could have accomplished.

